# Dissemination and prevalence of plasmid-mediated high-level tigecycline resistance gene *tet* (X4)

**DOI:** 10.3389/fmicb.2022.969769

**Published:** 2022-09-29

**Authors:** Shaqiu Zhang, Jinfeng Wen, Yuwei Wang, Mingshu Wang, Renyong Jia, Shun Chen, Mafeng Liu, Dekang Zhu, Xinxin Zhao, Ying Wu, Qiao Yang, Juan Huang, Xumin Ou, Sai Mao, Qun Gao, Di Sun, Bin Tian, Anchun Cheng

**Affiliations:** ^1^Avian Disease Research Center, College of Veterinary Medicine, Sichuan Agricultural University, Chengdu, China; ^2^Institute of Preventive Veterinary Medicine, Sichuan Agricultural University, Chengdu, China; ^3^Key Laboratory of Animal Disease and Human Health of Sichuan Province, Sichuan Agricultural University, Chengdu, China; ^4^Mianyang Academy of Agricultural Sciences, Mianyang, China

**Keywords:** antibiotic resistant bacteria, tigecycline resistance gene, plasmid-mediated, tet(X4), transmission, one health

## Abstract

With the large-scale use of antibiotics, antibiotic resistant bacteria (ARB) continue to rise, and antibiotic resistance genes (ARGs) are regarded as emerging environmental pollutants. The new tetracycline-class antibiotic, tigecycline is the last resort for treating multidrug-resistant (MDR) bacteria. Plasmid-mediated horizontal transfer enables the sharing of genetic information among different bacteria. The tigecycline resistance gene *tet*(X) threatens the efficacy of tigecycline, and the adjacent IS*CR2* or IS*26* are often detected upstream and downstream of the *tet*(X) gene, which may play a crucial driving role in the transmission of the *tet*(X) gene. Since the first discovery of the plasmid-mediated high-level tigecycline resistance gene *tet*(X4) in China in 2019, the *tet*(X) genes, especially *tet*(X4), have been reported within various reservoirs worldwide, such as ducks, geese, migratory birds, chickens, pigs, cattle, aquatic animals, agricultural field, meat, and humans. Further, our current researches also mentioned viruses as novel environmental reservoirs of antibiotic resistance, which will probably become a focus of studying the transmission of ARGs. Overall, this article mainly aims to discuss the current status of plasmid-mediated transmission of different *tet*(X) genes, in particular *tet*(X4), as environmental pollutants, which will risk to public health for the “One Health” concept.

## Introduction

The discovery of antibiotics is a milestone event in human medicine. With the large-scale use of antibiotics, while reducing the morbidity and mortality of bacterial infections, strains carrying different antibiotic resistance genes (ARGs) appeared and spread rapidly (Davies and Davies, 2010; Ahmad and Khan, 2019). The global sales of antimicrobials are estimated to reach 104,079 tons in 2030, an increase of 11.5% since 2017 (Tiseo et al., 2020). Antimicrobial resistance (AMR) is one of the public health issues of widely concern around the world, and ARGs are regarded as new environmental pollutants (Plantinga et al., 2015; Zhang et al., 2020c).Tetracycline have many desirable properties of antibiotics, such as their excellent anti-bacterial activity and oral benefits. They have been widely used in the treatment of human and animal infections or as animal growth-promoting feed additives (Roberts, 2003). However, only a small part of tetracycline can be absorbed after entering the body, and more than 75% of tetracycline will be excreted in the form of a prototype or metabolite (Liao et al., 2021).

Tigecycline belonged to tetracycline-class drugs, is a new class of glycylcycline antibiotics, approved by the FDA in 2005 (Wenzel et al., 2005; Stein and Babinchak, 2013; Hirabayashi et al., 2021). It has broad-spectrum anti-bacterial activity, especially against multidrug-resistant (MDR) gram-negative bacteria (Zha et al., 2020). Tigecycline is also considered as a drug of last resort to combat bacterial infections, and which is mainly used for the treatment of infections within skin tissue, anti-tumor, bacterial pneumonia, and complex intra-abdominal (Olson et al., 2006; Kaewpoowat and Ostrosky-Zeichner, 2015; Zhao et al., 2021). Furthermore, it is a third-generation tetracycline-class antibiotic, which was improved by adding a 9-tert-butyl-glycylamido side-chain modification structure to the central framework of minocycline, and thereby forming a steric hindrance, overcoming normal mechanisms of resistance to tetracyclines, such as parts of the efflux pump mechanism[*tet*(A-E), *tet*(K)] and ribosome protection mechanism[*tet*(M)] (Chopra, 2002; Livermore, 2005; Linkevicius et al., 2016). Tigecycline can act on bacterial ribosomes and inhibit bacterial protein synthesis by interfering with aminoacyl-tRNA binding to ribosomes (Chopra and Roberts, 2001). We have gathered, appraised, and reviewed the accessible relevant literature from online sources, including Science Direct, PubMed, and Google Scholar. The keywords were included but not limited to *tet*(X) genes, *Escherichia coli* (*E. coli*), IS*CR2*, IS*26*, antibiotic resistant bacteria (ARB), AMR, ARGs, MDR, plasmids, environmental pollutants, public health, resistance contact, clinical and veterinary settings. Moreover, the cited references were also explored for further referencing. This article summarized the mechanisms of tigecycline resistance and the prevalence of the plasmid-mediated high-level tigecycline resistance gene *tet*(X4) among the environment, animals, and humans. In addition, the origin of the *tet*(X) and the importance of mobile genetic elements (MGEs) during the dissemination of the *tet*(X) are discussed. The purpose of this article is to collect and organize the information available so far in one platform, and to provide a bridge for readers to understand that the prevalence of plasmid-mediated high-level tigecycline resistance genes, which can contaminate the natural environment, and further risking to public health. Moreover, we also made a positive outlook for the transmission of ARGs by viruses.

## Mechanism of tigecycline resistance

**Figure 1:**
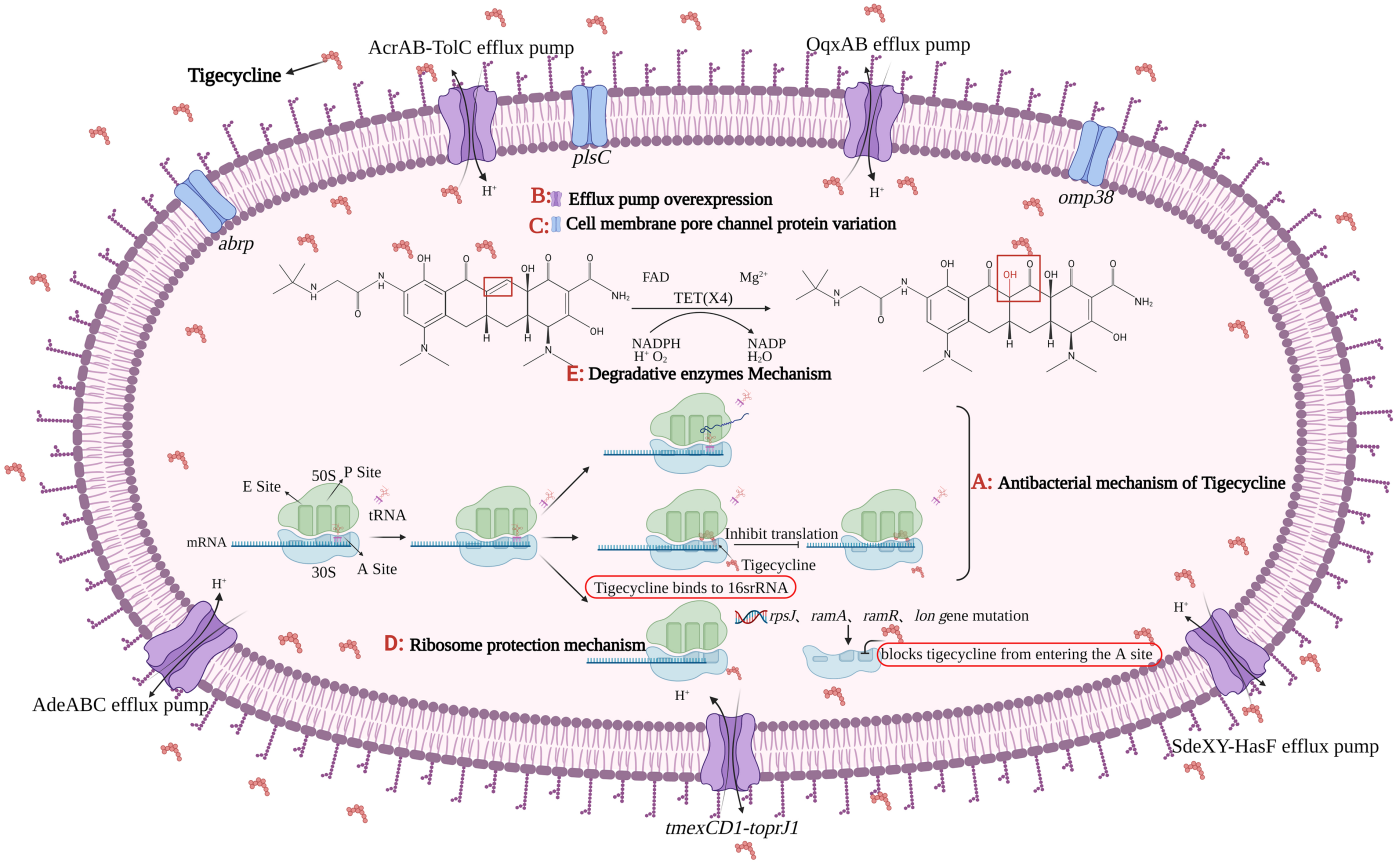
The figure was created with “BioRender.com” showing antibacterial mechanism and drug resistance mechanism of Tigecycline. **(A)** Tigecycline can act on bacterial ribosomes. After entering bacteria, tigecycline reversibly binds to the 16S rRNA in the 30S subunit of the ribosome, preventing tRNA from entering the A site, which eventually inhibits the process of transcription and translation in protein synthesis. There are four main tigecycline resistance mechanisms: **(B)** efflux pump overexpression; **(C)** cell membrane porin mutation; **(D)** ribosomal protection and **(E)** degrading enzyme mechanism. Among them, the expression product of plasmid-mediated *tet*(X4) gene belongs to the core member of degradative enzyme mechanism, and the Tet(X4) can catalyze the selective hydroxylation of tigecycline in the presence of FAD, Mg^2+^, O_2_ and NADPH, thus making tigecycline ineffective.

At present, the main mechanisms of bacterial resistance to tigecycline are efflux pump mechanism, cell membrane pore channel protein variation, ribosome protection mechanism, and drug-degrading enzyme mechanism (Figure 1).

### Efflux pump mechanism

An active efflux pump is a protein transport system of bacteria, it can excrete antibiotics entering the bacteria from itself, reducing antibiotic concentration in bacteria, so as to promote the growth of ARB (Venter et al., 2015; Bankan et al., 2021). There are five main efflux pump families involved in the active efflux of antibiotics, one is the ATP binding cassette (ABC) superfamily, which is the “primary active” transporter that directly uses ATP binding and hydrolysis to drive the free efflux of drugs (Rempel et al., 2019). The other four families are secondary active transport proteins, which are energy-acquiring transporters with proton pumps, including the major facilitator super (MFS) family, multidrug and toxic compound extrusion (MATE) family, small multidrug resistance (SMR) family, and resistance modulation division (RND) superfamily (Kumar et al., 2016; Lamut et al., 2019). In Gram-negative bacteria, overexpression of MFS family and RND family efflux pumps plays a significant role in tigecycline resistance, such as Tet(A), AcrAB-TolC, OqxAB, and AdeABC (Ruzin et al., 2007; Zhong et al., 2014; Chen et al., 2017), Tet(A) and AcrAB-TolC efflux pumps have been studied relatively comprehensively (Munita and Arias, 2016), their coding genes can be located on chromosomes or plasmids and can be transmitted *via* plasmids or transposons (Sheykhsaran et al., 2019). As a tetracycline efflux pump gene, *tet*(A) has no effect on tigecycline sensitivity (Fluit et al., 2005), but studies showed the double frameshift mutation of *tet*(A) can make strains resistant to tigecycline at a low level (Hentschke et al., 2010; Akiyama et al., 2013). A new RND type efflux pump gene cluster, named *tmexCD1-toprJ1*, was first identified in *Klebsiella pneumoniae* (*K. pneumonia*) in 2020. *TmexCD1-toprJ1* is widely present in *K. pneumoniae*, leading to a 4–32 fold increase in the minimal inhibitory concentration (MIC) of *K. pneumoniae* to tigecycline and eravacycline (Lv L. et al., 2020).

### Cell membrane porin variation

The 1-acyl-3-glycerol phosphatidyl transferase encoded by the *plsC* gene is located on the cell membrane of *E. coli*, and its primary function is to catalyze the synthesis of phospholipids, and then participate in the biosynthesis of bacterial cell membranes (Lu et al., 2005). By inducing *Acinetobacter baumannii* (*A. baumannii*) to be resistant to tigecycline, the researchers performed whole-genome sequencing analysis of the strains before and after induction, and found three factors that could reduce the sensitivity of tigecycline, which were the frameshift mutation of *plsC* and *omp38* as well as SNP synonymous mutation (Li et al., 2015). A new *abrp* gene was found in *A. baumannii*, which encodes the C13 family of peptidases and makes the bacteria less sensitive to tigecycline (Li et al., 2016).

### Ribosome protection mechanisms

The *rpsJ* gene can encode the production of the ribosomal structural protein S10. When there is a 12 bp deletion in *rpsJ*, the amino acid Rath at positions 53–56 of the S10 protein will be removed, resulting in a change in the binding site of tigecycline and bacteria, making bacteria resistant to tigecycline (Beabout et al., 2015; Bender et al., 2020). In addition to the S10 protein, mutations in the S3 and S13 proteins can also make bacteria resistant to tigecycline (Lupien et al., 2015). In *K. pneumoniae*, mutations in the *ramR* operon, *ramA*, *lon*, and *rpsJ* genes result in decreasing bacterial sensitivity to tigecycline (Fang et al., 2016). Mutation of *rpsJ* in *Enterococci* also leads to resistance to tigecycline (Cattoir et al., 2015). Mutations in the *rff*, *ropB* and *adeS* genes in *A. baumannii* can affect the normal function of the ribosome and thus confer tigecycline resistance to the strain (Hua et al., 2021).

### Mechanism of drug enzymatic degradation

Tet(X) is a FAD-dependent monooxygenase that regioselectively hydroxylates tetracycline substrates, leading to the non-enzymatic breakdown of an unstable compound (Ghosh et al., 2015). Tet(X) can only produce effect in the presence of FAD, NADPH, Mg^2+^, and O_2_ at the same time (Moore et al., 2005). Researchers proved that tigecycline was a substrate of Tet(X) by X-ray crystallography (Volkers et al., 2011), and in fact, Tet(X) can effectively degrade almost all tetracycline antibiotics, making bacteria resistant to tetracycline (Ghosh et al., 2015; Xu et al., 2022). *Tet*(X) gene was originally isolated from the anaerobic bacteria *Bacteroides fragilis* (Speer et al., 1991), however, according to recent reports, *tet*(X) appeared in *Riemerella anatipestifer* (*R. anatipestifer*) as early as the 1860s (Zhang et al., 2021a). In 2004, the *tet*(X) gene and its variant *tet*(X2) were discovered in anaerobic *Bacteroides*, then pointing out Tet(X) can degrade tigecycline, although it showed low levels of resistance to tigecycline, this phenomenon would still exist when *tet*(X) was transferred into *E. coli* (Guiney et al., 1984; Yang et al., 2004). Various *tet*(X) gene variants mediate different levels of tigecycline resistance. Compared with the Tet(X-X7), the enzymatic activity of the Tet(X4) has increased significantly. Researchers found five key residues (H231, M372, E43, R114, D308) could affect Tet(X4) enzyme activity in the tetracycline and FAD binding regions of the Tet(X4) (Xu et al., 2019). Subsequently, a new study has identified five mutants (L282S, A339T, D340N, V350I and K351E) in the structural domain of Tet(X2) when compared to Tet(X4), and demonstrated that the MIC of tigecycline increased 2–8 folds, when these five amino acid residues were mutated in the Tet(X2)-producing strain (Cui et al., 2021).

The plasmid-mediated tigecycline resistance genes *tet*(X3) and *tet*(X4) were first isolated from animal samples in 2019, which mediate high levels of antibiotic resistance to tigecycline, the MIC value can reach 32–64 mg/l (He et al., 2019). *Tet*(X4) is most commonly found in mobile plasmids and occasionally in chromosomes (Sun J. et al., 2019, 2020; Li et al., 2020b). Since the report of *tet*(X3/4), the degradative enzyme mechanism has gained more and more attention (He et al., 2019; Xu et al., 2022). At present, bismuth drugs and plumbagin can be used as Tet(X) inhibitors to improve the sensitivity of strains to tigecycline, which provides a new therapeutic strategy for the treatment of tigecycline-resistant bacterial infections (Deng et al., 2022; Xu et al., 2022).

## Origin and spread of *tet*(X4)

Although, the *tet*(X) gene was first isolated from the anaerobic *Bacteroidetes*, the current study points the origin of the *tet*(X) to *R. anatipestifer*, the *tet*(X) and its variants share the same ancestry with the monooxygenase gene carried in the chromosomes of *Flavobacteriaceae* bacteria. In Zhu’s study, 170 of 212 strains of *R. anatipestifer* carried the *tet*(X) gene (Zhu et al., 2018). Among 6,692 strains isolated from 13 different hospitals, almost all of the *tet*(X)-positive strains belonged to the *Flavobacteriaceae*. They then performed a phylogenetic analysis of the different evolutionary patterns of *tet*(X), in which one of the pathways involving the *Flavobacteriaceae* produced a major evolutionary branch, suggesting that it can be considered as the potential ancestral source of *tet*(X) (Zhang et al., 2020a). Umar et al. collect 57 non-repetitive sequences of *R. anatipestifer* in GenBank, of which *tet*(X) gene was detected in 47 genomes, and they have high similarity when compared with *tet*(X4) gene (Umar et al., 2021). The same finding was also reported in other study (Cui et al., 2021). When analyzing the evolutionary trajectory of the *tet*(X) gene, they found that most of the *tet*(X)-positive strains belonged to the *Flavobacteriaceae*, it has a higher detection rate than other species and is widely distributed in different clades of *tet*(X). Their latest study also inferred that the *tet*(X) gene originated in *Flavobacteriaceae* and can be transmitted to environmental and clinical strains such as *E. coli* and *Acinetobacter* with the help of the mobilization of IS*CR2* element (Chen et al., 2020).

The MGE such as IS*CR2* and IS*26* are essential for the spread of *tet*(X) gene. A 4608 bp element consisting of an IS*CR2*, a *tet*(X4) and a partner gene *catD* forms a canonical RC transposable unit (RC-TU) mediated by IS*CR2*, of which the 2,760 bp element of *catD*-*tet*(X4) is highly conserved. When transposition occurs, the IS*CR2*-*catD*-*tet*(X4)-IS*CR2* composite transposon structure is often generated, and the upstream or downstream of IS*CR2* element may be inserted and truncated by other IS elements, such as IS*26* (Chen et al., 2021; Liu et al., 2022). In addition, only single-copy IS*CR2* elements was sufficient to transpose adjacent DNA sequences through the process of rolling circle transposition (Poirel et al., 2009; Partridge et al., 2018). IS*26* was also often found in plasmids resistant to antibiotics, and it can participate in the progress of plasmid fusion and gene recombination (He et al., 2015; Du et al., 2020; Li et al., 2020b), and IS*26* can also be inserted into both ends of RC-TU, allowing IS*CR2* residues-*tet*(X4) to spread through a novel transmission mechanism (Liu et al., 2022). It has been found that the IS*CR2* element is frequent adjacent to *tet*(X4) or other *tet*(X) variants, which suggests IS*CR2* is more likely to participate in spread of *tet*(X) variants (Wang L. et al., 2019; Liu et al., 2020; Fu et al., 2021). In a conserved genetic environment and uncertain transferability among different bacteria, the co-action of IS*CR2* and IS*26* may be the main driving forces for the widespread of *tet*(X4; Dai et al., 2022; Zhang et al., 2022).

## Prevalence of *tet*(X4)

**Figure 2:**
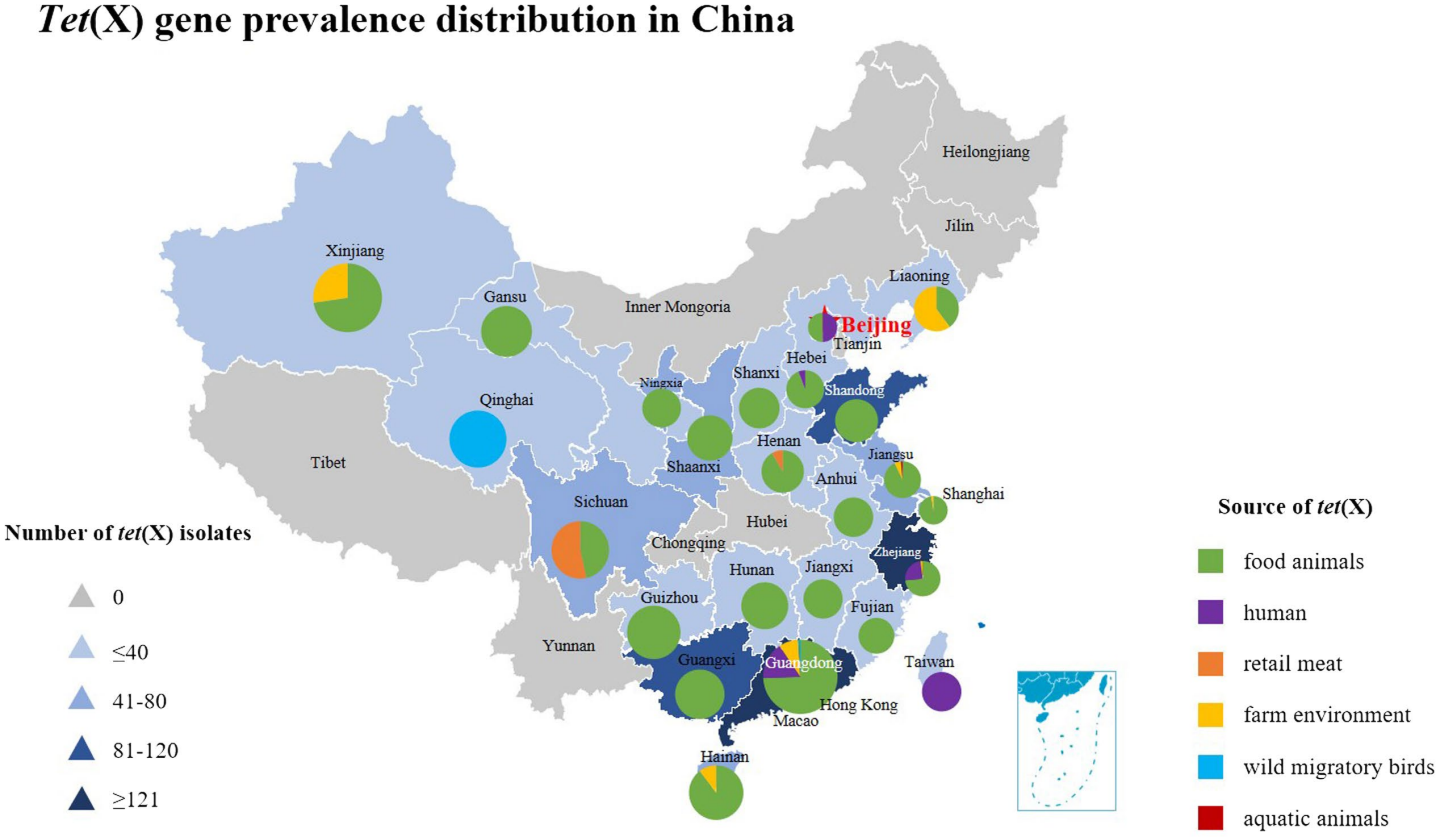
The distribution of *tet*(X) genes in different parts of China showing these genes have been found in 24 provinces from 2015 to 2022. The triangle in the figure indicates the number of *tet*(X)-positive strains isolated in each region of China, and the square indicates different sources of *tet*(X) genes, which corresponds to the pie chart in the figure. Furthermore, the different sources of *tet*(X) genes in the listed provinces can also be found, using color indication.

Tetracycline resistance genes speculated to be of environmental origin but are now widely distributed in commensal and pathogenic bacteria (Thaker et al., 2010). The extensive use of first or second-generation tetracycline-class drugs played a major role in the emergance of tetracycline resistance genes, especially oxytetracycline, chlortetracycline, and doxycycline (Aminov, 2021). Since the discovery of the plasmid-mediated high-level tigecycline resistance genes *tet*(X3/X4) in 2019, reports of *tet*(X) have gradually increased around the world (Table 1). *Tet*(X4)-positive strains have spread globally and have been detected in animals, humans and the environment, which largely limited the use of tigecycline (Xu et al., 2022). The *tet*(X) gene and its variants were present in 23 countries on six continents (Pan et al., 2020; Wang J. et al., 2021), which are also widely present in various bacterial species, including *R. anatipestifer*, *E. coli*, *Acinetobacter*, *K. pneumoniae*, *Salmonella*, *Proteus*, *La Providencia bacteria, Bacteroides bacteria*, *Pseudomonas bacteria*, and *Aeromonas caviae* (Chen et al., 2019a, 2020). Moreover, most of the *tet*(X4) genes are located on different types of plasmids such as IncQ1, IncX1, IncFIB, IncHI1, F-:A18:B-, ColE2-like, IncN, p0111 and hybrid plasmids (Fang et al., 2020), among which the IncX1 type is the most common (Cai et al., 2021; Cui et al., 2022). The Nomenclature Center^1^ recommends that only *tet*(X) will be used in the future, because the *tet*(X) gene variant DNA similarity is in the range of 83–100% among *tet*(X2)-*tet*(X14), corresponding amino acid similarity is between 82 and 100%, which is greater than the standard of 79% amino acid similarity. In this article, for the convenience of description, the previous classification method is still used. This article also summarizes the prevalence of *tet*(X) gene and its variants in China in recent years as shown in Figure 2.

**Table 1:** Global prevalence of different *tet*(X) genes in recent years.

Province/Country	Years of samples	Source (Reference or NCBI database)	Sample sources	*Tet*(X) types	Localization of gene	Plasmid types	Sequence types	*Tet*(X)-positive isolates	ESBLs/*mcr* genes	Bacterial strains
Sichuan	2018–2020	(Bai et al., 2019; Sun C. et al., 2019, 2020; Li et al., 2021a; Tang et al., 2021; Feng et al., 2022)	Food animals	*tet*(X4)	Plasmid	IncQ1-IncY IncX1	ST48, ST4541, ST9772, ST972, ST410, ST10, ST195, ST3696, ST25, ST196	27	*cfr* *mcr-1* *bla*_TEM-1B_	*E. coli* *Citrobacter freundii*
		(Li, 2020a; Lv H. et al., 2020; Sun et al., 2021b)	Retail meat	*tet*(X4)	Plasmid	IncFIA-IncHI1A-IncHI1B IncX1	ST4656, ST1788, ST871, ST48, ST1638, ST542, ST877, ST641, ST10, ST3858, ST195, ST515	31	*bla*_NDM-5_ *bla*_SHV-12_ *bla*_CTX-M-55_ *bla*_CTX-M-14_	*E. coli*
Guangdong	2016–2019	(He et al., 2019; Sun C. et al., 2019,Sun J. et al., 2019; Chen et al., 2020; Cheng et al., 2020; Cui et al., 2020; Sun et al., 2020; Zheng et al., 2020; Chen et al., 2021; Li et al., 2021a; Yu et al., 2021; Wu et al., 2022)	Food animals	*tet*(X/X2) *tet*(X3) *tet*(X4) *tet*(X5) *tet*(X6) *tet*(X14)	Plasmid Chromosome	IncFIA-IncHI1A-IncHI1B	ST4535, ST10, ST23, ST215, ST206, ST789, ST1196, ST2144, ST195, ST101, ST109, ST789, ST2064, ST980, ST355, ST542, ST8302	236	*bla*_TEM-1B_ *bla*_NDM-1_ *bla*_OXA-58_	*E. coli* *Acinetobacter* *Citrobacter freundii* *Enterococcus faecalis* *Enterobacter cloacae*
		(Chen et al., 2019a; Cui et al., 2020; Sun et al., 2020; Wang Y. et al., 2020; Zheng et al., 2020; Chen et al., 2021; Yu et al., 2021; Gao et al., 2022)	Farm environment	*tet*(X) *tet*(X3) *tet*(X4) *tet*(X6)	Plasmid Chromosome	IncFIA-IncHI1A–IncHI1B	ST645, ST10, ST37	28	*bla*_SHV-81_ *bla*_SHV-110_	*Acinetobacter* *E. coli* *K. pneumoniae* *Aeromonas cavive*
		(Chen et al., 2019b)	Wild migratory birds	*tet*(X4)	Plasmid Chromosome	F-:A18:B- IncHI1	ST1196, ST6833, ST641	3	–	*E. coli*
		(Chen et al., 2020; Wang Y. et al., 2020; Cui et al., 2022)	Human	*tet*(X3) *tet*(X4)	Plasmid	IncX1, IncFIA, IncHIA, IncHIB	ST10, ST48, ST877, ST2144, ST101, ST515, ST542, ST871, ST4456, ST38, ST137, ST201, ST7176, ST10548, ST6984, ST46, ST1249, ST195, ST155, ST58, ST4014, ST7686, ST1114, ST7450, ST1684	51	*mcr-5.2* *bla*_NDM_ *bla*_OXA_ *bla*_TEM_ *bla*_SHV_ *bla*_CTX-M_	*E. coli* *Acinetobacter*
Jiangsu	2015–2020	(He et al., 2019; Sun J. et al., 2019; Chen et al., 2020; Peng et al., 2020; Li et al., 2020b; He T. et al., 2020; Li et al., 2020c; Yu et al., 2021; Cheng et al., 2021a; Li et al., 2021b)	Food animals	*tet*(X3) *tet*(X4) *tet*(X6) *tet*(X15)	Plasmid Chromosome	IncHI1, IncFIB(K), IncX1, IncA/C2	ST3997, ST284, ST93, ST1286, ST155, ST327, ST1459, ST48, ST3944, ST10170, ST8302	137	*bla*_CTX-M_ *cfr* *bla*_NDM-1_ *bla*_TEM − 1B_	*E. coli* *Acinetobacter* *Proteus* *Citrobacter freundii Providencia*
		(Li et al., 2020b; Yu et al., 2021)	Farm environment	*tet*(X4)	Plasmid	–	–	21	–	*E. coli*
		(Li et al., 2019)	Aquatic animal	*tet*(X2/3.2)	Plasmid	–	–	1	–	*Brevibacterium brevis*
Shanghai	2015–2019	(Chen et al., 2020; Sun et al., 2020; Wang J. et al., 2020; Li et al., 2021a; Wang J. et al., 2021)	Food animals	*tet*(X) *tet*(X3) *tet*(X)	Plasmid	IncFIA18-IncFIB(K)-IncX1 IncX1, IncQ	ST761, ST165, ST195, ST295, ST2144	41	*bla*_OXA-58_	*E. coli* *Acinetobacter* *K. pneumoniae*
		(Wang J. et al., 2021)	Farm environment	*tet*(X)	Chromosome	–	–	1	–	*Proteus*
Henan	2013–2019	(Sun C. et al., 2019, 2020; Li et al., 2020d; Li et al., 2021a)	Food animals	*tet*(X4) *tet*(X6)	Plasmid Chromosome	IncX1 IncFIA-IncFIB(K)-IncX1	ST10, ST48, ST641, ST2345	11	*mcr-1*	*E. coli*
		(He D. et al., 2020)	Retail meat	*tet*(X6)	–	–	–	1	–	*Proteus*
Hebei	2019	(Li et al., 2021a)	Food animals	*tet*(X4)	Plasmid	IncX1, IncQ, IncFIA-IncHI1A-IncHI1B	ST48, ST10, ST4156, ST195, ST6833, ST515, ST2064, ST58	16	–	*E. coli* *K. pneumoniae*
	2017	(Wang L. et al., 2019)	Human	*tet*(X5)	Plasmid	–	–	1	–	*Acinetobacter*
Shandong	2017–2019	(Bai et al., 2019; He et al., 2019; Cui et al., 2020; Du et al., 2020; Liu et al., 2020; Li et al., 2021a; Yu et al., 2021)	Food animals	*tet*(X/X2) *tet*(X3) *tet*(X4) *tet*(X6)	Plasmid Chromosome	IncFII, IncFIA-IncHI1B-IncHI1A	ST761, ST746, ST101, ST10, ST847	83	*bla*_TEM − 1B_ *bla*_CTX-M-55_	*Acinetobacter* *Myroides* sp. *E. coli* *K. pneumoniae* *Proteus*
Zhejiang	2015–2019	(Chen et al., 2020; Zhang et al., 2020b; Li et al., 2021a; Cheng et al., 2021b; Zheng et al., 2022)	Food animals	*tet*(X2) *tet*(X3) *tet*(X4) *tet*(X6) *tet*(X5.2) *tet*(X14)	Plasmid Chromosome	IncFIA-IncHI1B-IncHI1A IncFIA-IncHI1B-IncX1	ST10, ST773, ST1196, ST6883, ST641, ST515, ST767	100	*bla*_OXA-58_ *bla*_NDM-1_	*Acinetobacter* *Enterococcus faecalis Proteus* *E. coli*
		(Cheng et al., 2021b)	Farm environment	*tet*(X2)	–	–	–	3	–	*Myroides* sp.
		(He et al., 2019; Ruan et al., 2020; Zeng et al., 2021)	Human	*tet*(X4)	Plasmid	IncX1	ST773	33	*mcr-1 bla*_CTX-M-14_	*E. coli*
Jiangxi	2015–2018	(Sun J. et al., 2019; Chen et al., 2020)	Food animals	*tet*(X4) *tet*(X3)	Plasmid Chromosome	IncQ1	ST761, ST515, ST871, ST8302	37	*mcr-1*, *bla*_CTX-M-14_	*E. coli* *Acinetobacter*
Hainan	2017–2018	(Chen et al., 2020; Cui et al., 2020)	Food animals	*tet*(X) *tet*(X3)	Plasmid	–	–	43	*bla*_NDM-1_	*Acinetobacter*
			Farm environment	*tet*(X)	Plasmid	–	–	5	*bla*_OXA-58_	*Acinetobacter*
Guangxi	2017–2020	(Sun J. et al., 2019; Cui et al., 2020; Feng et al., 2022)	Food animals	*tet*(X) *tet*(X4)	Plasmid	–	ST1196, ST10, ST1415, ST34, ST109, ST48, ST195, ST799, ST2223, ST1244, ST3888, ST6404, ST641, ST677, ST452, ST1250	97	–	*Acinetobacter* *E. coli*
Fujian	2018	(Sun J.et al., 2019; Chen et al., 2020; Cui et al., 2020)	Food animals	*tet*(X) *tet*(X4)	Plasmid	–	ST8302, ST761, ST515, ST8338	26	–	*Acinetobacter*
Qinghai	2015–2018	(Chen et al., 2020)	Wild migratory birds	*tet*(X4)	–	–	–	5	–	*Acinetobacter*
Xinjiang	2017–2018	(Cui et al., 2020)	Food animals	*tet*(X)	–	–	–	8	*bla*_NDM-1_	*Acinetobacter*
			Farm environment	*tet*(X)	–	–	–	3	–	*Acinetobacter*
Liaoning	2018	(Cui et al., 2020)	Food animals	*tet*(X)	–	–	–	2	–	*Acinetobacter*
			Farm environment	*tet*(X)	–	–	–	3	–	*Acinetobacter*
Taiwan	2019–2020	(Hsieh et al., 2021; Wang et al., 2021a)	Human Environment	*tet*(X) *tet*(X10)	Chromosome	–	ST793, ST723	7 1	*bla*_OXA-72_	*Acinetobacter* *Amniculibacterium aquaticum*
Shanxi	2018–2020	(Li et al., 2021a; Feng et al., 2022)	Food animals	*tet*(X4)	Plasmid	IncFIA-IncHI1B-IncHI1A IncX1	ST641, ST58, ST515, ST2064, ST6833, ST10, ST48, ST4156	11	–	*E. coli*
Gansu	2019	(Li et al., 2021a)	Food animals	*tet*(X4)	Plasmid	IncFII	ST540	1	–	*E. coli*
Anhui	2019	(Li et al., 2021a)	Food animals	*tet*(X4)	Plasmid	IncFIA-IncHI1B-IncHI1A IncFIA-IncFIB-IncX1 IncX1, IncFII	ST877, ST2035, ST218	8	–	*E. coli*
Beijing	2018	(Zhai et al., 2022)	Human	*tet*(X4)	Plasmid	IncFIIK	ST534	1	–	*K. pneumoniae*
		(Sun et al., 2020)	Food animals	*tet*(X4)	Plasmid	IncFIA-IncHI1B-IncHI1A	ST744	1	–	*E. coli*
Shaanxi Ningxia	2018–2020	(Sun et al., 2020; Feng et al., 2022)	Food animals	*tet*(X4)	Plasmid	IncX1, IncN, IncR, IncY, IncFIA, IncFIB	ST877, ST2035, ST10392, ST10, ST7366, ST890, ST3580, ST442, ST278, ST4429, ST1602, ST746, ST48, ST189, ST8504, ST1437, ST7604	7,346	–	*E. coli*
Guizhou	2018	(Sun et al., 2020)	Food animals	*tet*(X4)	Plasmid	–	ST48, ST202, ST542, ST206, ST890	1	–	*E. coli*
Hunan	2015–2018	(Chen et al., 2020)	Food animals	*tet*(X3)	Plasmid	–	–	14	–	*Acinetobacter*
Vietnam	2021	(Dao et al., 2022)	River	*tet*(X4)	Chromosome	–	–	1	*bla*_OXA-48_	*Shewanella Xiamen*
Sierra Leone	2010–2011	(Leski et al., 2013)	Human	*tet*(X)	–	–	–	11	–	*Enterobacter cloacae E. coli* *K. pneumoniae* *Pseudomonas* *Delftia acidovorans* *Comamonas testosteroni*
Singapore	2018	(Ding et al., 2020)	Human	*tet*(X4)	Plasmid	IncI1	ST73	2	*mcr-1*	*E. coli*
Japan	2012	(Usui et al., 2021)	Food animals	*tet*(X6)	Plasmid	IncW	–	1	–	*E. coli*
Chile	2010–2021	(Concha et al., 2021; Wang et al., 2021a)	Aquatic animals	*tet*(X) *tet*(X10)	–	–	–	3	–	*Epilithonimonas* *Chryseobacterium* sp.
Pakistan	2018–2019	(Mohsin et al., 2021; Li et al., 2022)	Food animals Farm environment Human	*tet*(X4) *tet*(X7)	Plasmid	IncFII, IncQ	ST6726, ST694, ST4388、ST224	41 1	*mcr-1*	*E. coli* *Pseudomonas aeruginosa*
United Kingdom	1966–2020	(Martelli et al., 2022)	Food animals Human Rainbow trout	*tet*(X4) *tet*(X12) *tet*(X4) *tet*(X7) *tet*(X6)	Plasmid – – –	IncX1-IncY – – –	ST1140 – – –	1 1 5 2 2	–	*E. coli* *Riemerella anatipestifer* *Salmonella* *Shigella soneii* *Enterobacter hormaechei* *Salmonella Typhimurium* *Chryseobacterium* sp.
Norway	–	(Marathe et al., 2021)	Wastewater treatment plants	*tet*(X4)	Plasmid	IncFIA/FIB	ST167	1	*bla*_CTX-M-14_	*E. coli*
Belgium	2007–2017	LDIS01000001.1 SELG01000025.1	Food animals Musca domestica	*tet*(X10)	– –	– –	– –	1 1	– –	*Arcobacter thereius* *Apibacter muscae*
South Africa	2013	MKSZ01000121.1	Thiocyanate stock biobioreactor	*tet*(X10)	–	–	–	1	–	*Bacteroidales bacterium*
United States of America	2010–2018	(Wang et al., 2021a)	Human Environment	*tet*(X10) *tet*(X7) *tet*(X10)	– – –	– – –	– – –	47 1 2	– – –	*Bacteroides* sp. *E. coli* *Chryseobacterium* sp. *Bacteroides* sp.
Australia	2018	VSOP01000024.1	Mus musculus	*tet*(X10)	–	–	–	1	–	*Alistipes* sp.
Ireland	2017	VLSQ01000048.1 VLSR01000042.1 SMTB01000142.1	Environment Food animals	*tet*(X3) *tet*(X6)	– –	– –	– –	2 1	– –	*Acinetobacter* sp.
Bolivia	2016	PQTA01000018.1	Human	*tet*(X7)	–	–	–	1	–	*E. coli*
Turkey	2021	(Kürekci et al., 2022)	Wastewater	*tet*(X4)	Plasmid	IncFIA-IncHI1-IncFIB(K)	ST609	2	*bla*_SHV-12_	*E. coli*

### Prevalence of *tet*(X4) in animals

**Figure 3:**
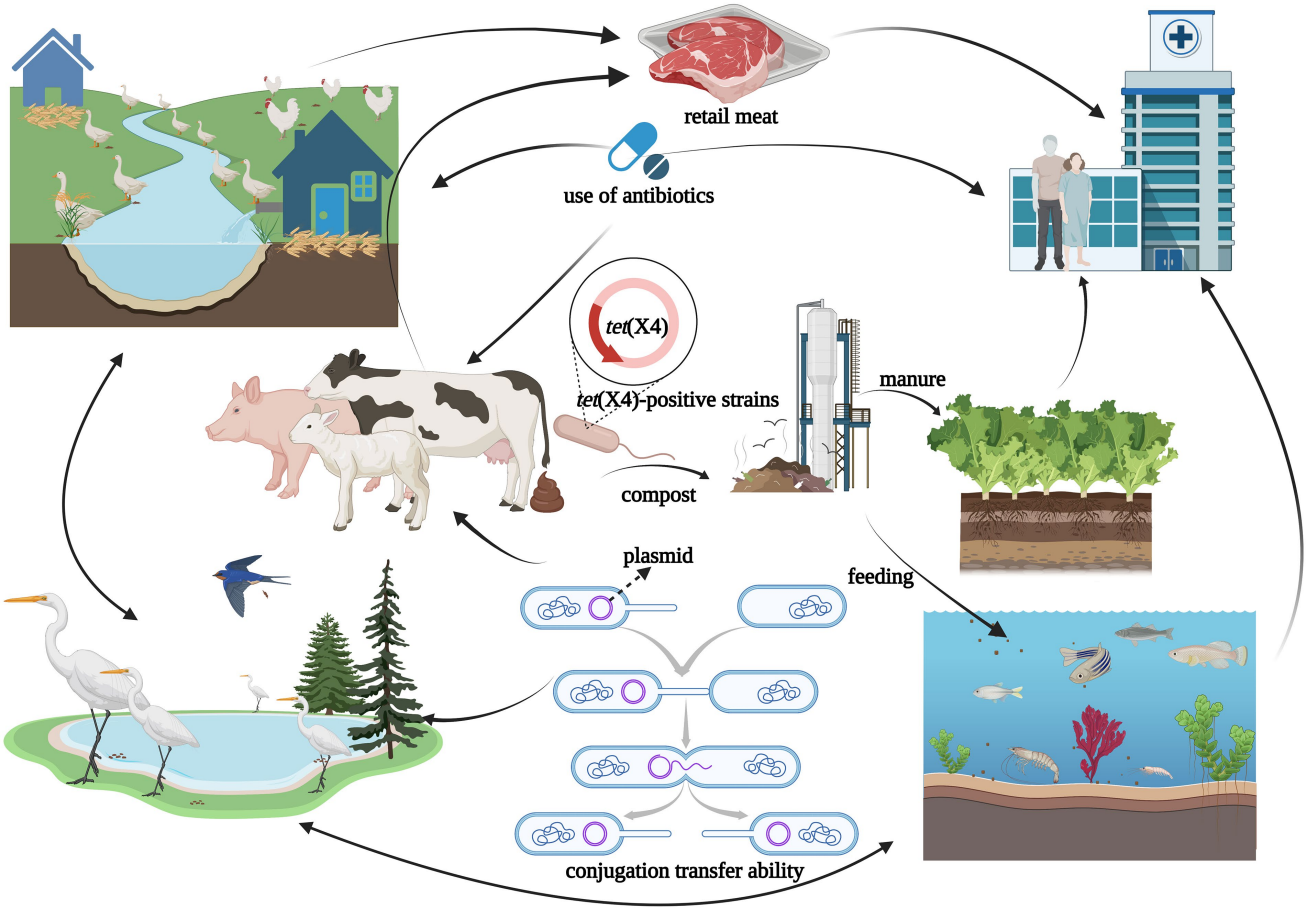
The figure was created with “BioRender.com” showing transmission routes illustration of *tet*(X4)-positive strains in natural environment. Possible dissemination routes of *tet*(X4)-positive strains showed by arrows among different reservoirs such as ducks, geese, migratory birds, chickens, pigs, cattle, aquatic animal, agricultural field, meat, and humans. The horizontal transmission of *tet*(X4) among reservoirs risked to public health for the “One Health” concept.

Antibiotics are commonly used in livestock production to maintain animal health and productivity. However, the absorption of antibiotics in the body is low, and most of them are excreted in the form of metabolites with feces and urine (Qiu et al., 2016). The antibiotic residues and ARGs carried in animal feces can be transmitted to the environment or humans, showing a potential source of ARGs (Ji et al., 2012; Van Boeckel et al., 2015). Tigecycline is currently approved for Human clinical use only, but the *tet*(X4) gene has been detected in food animals, retail meat, aquatic animals, and wild animals (Figure 3). Moreover, *tet*(X4) is currently detected in isolates from various animal origin samples, including pigs, ducks, geese, chickens, cattle, freshwater fish and shrimp, and migratory birds, with pig sources in particular predominating (Table 1). In a study based on a metagenomics approach, it was shown that among the abundant of ARGs in pig manure and its receiving environment (sewage, crops, soil, etc.), the tetracycline resistance genes were prevalent in pig farms (Tong et al., 2022). The same is true for pig slaughterhouses, suggesting that *tet*(X4)-carrying plasmids play an essential role in the spread of this drug related ARGs (Li et al., 2020b). Worth noting that the first isolation of plasmid mediated-*tet*(X4) was also obtained from the pig-derived sample (He et al., 2019). So far, 24 provinces in China have reported the emergence of *tet*(X), with Guangdong, Zhejiang, and Shandong having the largest number of positive strains (Figure 2). Li et al. (2021c) isolated 32 *tet*(X4)-positive strains from feces and anal swabs of pigs in Shanxi. At the same time, *tet*(X4)-positive *E. coli* were also detected in the sewage and soil of the pig farm environment. These isolates have different ST types, but their *tet*(X4)-carrying plasmids have the same replicon type, indicating that these plasmids are transferred horizontally among different reservoirs, and horizontal transfer maybe the main way for *tet*(X4) to spread in the surrounding environment (Sun J. et al., 2019). During 2016–2018, researchers isolated the *tet*(X)-positive *Acinetobacter* from pig, chicken, duck and goose feces in multi-regional farms of seven provinces, China (Guangdong, Hainan, Guangxi, Fujian, Shandong, Xinjiang, and Liaoning; Cui et al., 2020). Zhang et al. have detected 51 (17%) *tet*(X)-positive strains from 296 rectal swabs of healthy dairy cows, including the strains of *tet*(X3)-positive *Acinetobacter* and *tet*(X4)-positive *E. coli* (Zhang et al., 2020b). The prevalent range of *tet*(X) continues to expand, *tet*(X) and its variant genes have been detected in different reservoirs, and *tet*(X)-carrying plasmids have high mobility, which can be transmitted horizontally among different species.

The co-existence of *tet*(X4) with other important ARGs is noteworthy. Specifically, the *tet*(X) gene co-existed with the *flor* gene in most cases, the latter encoding chloramphenicol efflux pumps, which can be also co-transferred (Du et al., 2004; Fu et al., 2021). Further, ESBL genes and colistin resistance genes often co-existed with *tet*(X4) in *Enterobacteriaceae* (Table 1). In a retrospective study, five pig-derived *tet*(X4)-positive strains were detected in Sichuan, Henan, and Guangdong of China, and two of these *tet*(X4)-positive *E. coli* also carried the *mcr-1* gene (Sun C. et al., 2019). Tang et al. (2021) found eight *tet*(X4)-positive strains in two commercial pig farms in Sichuan, and three of them co-existed with the *cfr* gene in *E. coli*, and both ARGs were located on a novel hybrid plasmid, which could be transferred to the recipient bacteria. Li et al. (2020c) screened one strain of *tet*(X4)-positive *E. coli* and two strains of *tet*(X6)-positive *aspergillus* in different chicken farms, while the *tet*(X6) gene co-existed with the carbapenem resistance gene *bla*_NDM-1_. The same situation also existed in other country, where the *tet*(X4) gene was detected to co-exist with the colistin resistance gene in Pakistan (Mohsin et al., 2021; Li et al., 2022). Specifically, Li et al. (2022) detected 36 *tet*(X4)-positive strains, of which 24 *tet*(X4)-positive strains co-carried the *mcr-1* gene. Mohsin et al. (2021) detected four *tet*(X4)-positive *E. coli* from farm animals and slaughterhouse effluents, and three *E. coli* contained the *mcr-1.1* gene. It should be noted that the resistance to tigecycline or colistin can be transferred by the transmission of plasmids, which posed an enormous threat to the clinical treatment of MDR bacterial infections (Ruan et al., 2020; Xu et al., 2021; Zhang et al., 2021b).

Food animals such as pigs and poultry are the primary source of high-quality protein for humans (Henchion et al., 2014), they have been slaughtered in slaughterhouses before entering the market, and *tet*(X) has also been detected in retail meat, which indicated that the slaughterhouse might be a potential reservoir for *tet*(X) (Homeier-Bachmann et al., 2021; Mohsin et al., 2021). There are also some reports on *tet*(X) from retail meat sources in Sichuan and Henan. In 2019, Sun et al. collected 311 retail meat samples from Sichuan province and detected 25 *tet*(X4)-positive *E. coli* strains, most of which were isolated from the raw pork (52%), chicken (40%), duck (4%), and beef (4%; Sun et al., 2021a). In addition, five *tet*(X4)-positive *E. coli* strains were isolated from retail chicken during routine monitoring of ARGs in the Sichuan market in 2020. Interestingly, one of the *tet*(X4)-carrying plasmids from retail chicken was 99% identity to the pig-derived *tet*(X4)-carrying plasmid, and others had the *tet*(X4) gene localized on hybrid plasmids (Lv H. et al., 2020). This phenomenon suggests that *tet*(X4)-carrying plasmids can spread among different animals, which lead to the dissemination of *tet*(X4) in the ecological environment.

In addition to food animals, tigecycline resistance genes have also been detected in wild animals. In 2018, Chen et al. (2019b) isolated three strains of *tet*(X4)-positive *E. coli* from the feces of migratory birds in Guangdong, two of which were located on the plasmid, and the remaining one was located on the chromosome. The *tet*(X4)-carrying plasmid isolated from the migratory birds had a high degree of similarity with one plasmid isolated from human samples. In addition, five *tet*(X4)-positive *Acinetobacter* were also isolated from the bar-headed goose samples in Qinghai (Chen et al., 2020). In the latest report, researchers also detected the *tet*(X) variant genes in wild fish and shrimp (Li et al., 2019; Concha et al., 2021). Wild animals were not directly exposed to clinical antibiotics, but more and more ARGs were detected in them, indicating wild animals including migratory birds, were likely to be involved in the large-scale exchange of ARGs, especially long-distance transmission of cross species (Allen et al., 2010; Wang et al., 2017; Zeballos-Gross et al., 2021; Luo et al., 2022).

### Prevalence of *tet*(X4) in humans

Tigecycline was approved for clinical use in 2005, and which was introduced in China in 2012. *Tet*(X) was detected in human clinical samples in 2013, with 11 *tet*(X) positive strains isolated from 52 samples, including stool, semen, blood, and urine in a Sierra Leonean hospital (Leski et al., 2013). Ding et al. (2020) conducted a retrospective screening study on 109 fecal samples, and detected *tet*(X4)-positive strains in the intestinal microflora of healthy human, with an isolation rate of 10.1%. Subsequently, *tet*(X4)-positive *E. coli* were also reported in clinical isolates from Guangdong, Hebei, Zhejiang, Beijing, Sichuan, and other places in China (Table 1). It can be seen that the *tet*(X) gene is not uncommon in hospital clinical isolates, and *tet*(X4) may be widely distributed in the human gut microflora, with great risk of transmission. In 2019, Cui et al. (2022) collected 1,001 stool samples from hospital inpatients in Guangdong Province of China, isolated 48 (4.8%) *tet*(X4)-positive *E. coli*. Notably, the hybrid plasmid was found to be prevalent in *tet*(X4)-positive strains of animal origin, with the characteristics of stable existence and horizontal transfer (Sun C. et al., 2019), which predicted this *tet*(X4)-carrying plasmid can be transmitted among humans, animals and the environment, thus facilitating the wide spread of *tet*(X4) in the ecosystem. The co-existence of *tet*(X4) with *mcr* and ESBL genes in the clinical setting is a great concern. Ruan et al. (2020) found one *E. coli* strain co-harboring *tet*(X4) with *mcr-1* on the same conjugative plasmid from the urine sample of a clinical patient in Zhejiang Province, China. Further, two *E. coli* strains carrying both *mcr-1* and *tet*(X4) were isolated in Singapore (Ding et al., 2020). Meanwhile, *bla*_CTX_, *bla*_OXA_, *bla*_NDM_, and *bla*_SHV_ genes were also detected to be co-existence with *tet*(X) in one strain (Table 1). Tigecycline and colistin are the last resort for treating MDR bacteria, and the co-existence of *tet*(X) with *mcr* and ESBL genes limited the choice of clinical antibiotics, which subsequently poses a significant threat to public health.

ARB are persistent pollutants in the environment in which humans are in close contact (Kim and Aga, 2007). ARB can be transmitted to other hosts through human activities when conditions are favorable (Allen et al., 2010). Except for the hospital clinical environment, the live poultry market (LPM) is also a vast reservoir of ARGs (Wang Y. et al., 2019; Wang et al., 2021b). The *tet*(X3) and *tet*(X4) genes have been detected in the intestinal flora of LPM workers and the surrounding environment (Wang Y. et al., 2020), which indicated that the plasmid-mediated tigecycline resistance gene might exist in LPM for a long period. The ARGs are likely to be transmitted from live poultry to LPM staff, ecological environments or other animals.

### Prevalence of *tet*(X4) in the environment

Antibiotics and ARGs were detected in various environments (Qiao et al., 2018). The humans, animals, and ecological environments are components of the “One health” concept, and they have important connections and can influence each other. Therefore, they can acquire ARGs through different pathways and achieve the flow of ARGs among different reservoirs (Anyanwu et al., 2021), including *tet*(X4) (Figure 3). In recent years, the environment has played an increasing role on the spread of antibiotic resistance (Finley et al., 2013; Bengtsson-Palme et al., 2014; Bondarczuk et al., 2016; Lerminiaux and Cameron, 2019). The ARGs and ARB existed in large numbers within the environment and can be transmit to reservoirs (Lin et al., 2021), such as rivers contaminated by animal manure, the soil around livestock farms, manure-irrigated agricultural fields, and sewage treatment plants. The abuse use of antibiotics and the spread of antibiotic resistance caused by animal husbandry is one of the main concerns of sustainable agriculture(Manyi-Loh et al., 2018), where the use of first or second-generation tetracycline-class drugs was high, with subtherapeutic dosing in the forage (Yezli and Li, 2012). In animal husbandry, a wider range of antibiotic options lead to the spread of ARGs in agriculture to the human microbiota (Aminov, 2011). Animal manure as the valuable renewable fertilizer was often applied to the cropland (Zhou X. et al., 2019; Lima et al., 2020), which was found to contain different ARB and ARGs. Moreover, water as a good transport route for nutrients and contaminants was also a major reservoir for ARGs (Vaz-Moreira et al., 2014; Manaia et al., 2016; Miłobedzka et al., 2022). Specifically, macrogenomic analysis of wetland effluents and sediments in the Yangtze Delta region revealed a high abundance of the *tet*(X) gene (Du et al., 2022). *Tet*(X) and their variants were detected in farm soil, manure, and lettuce samples near chicken farms in Jiangsu, Jiangxi, and Sichuan provinces of China, and even in soil samples far from these farms (He et al., 2021). Cui et al. (2020) collected samples from some poultry farms in seven provinces across China, where *tet*(X)-positive strains from sewage and soil were isolated at 7.5% and 6.7%, respectively, and *tet*(X) was detected to be localized on the same plasmid with *bla*_NDM-1_. These reports on identification and analysis of *tet*(X4) in the farm environment suggest that animal manure, sewage, and soil can influence with each other in this ecology. Moreover, *tet*(X4) can be transmitted among them, and the farm environment may be a massive reservoir of ARGs.

## Discussion and prospects

The phenomenon of MDR of bacteria is a significant concern worldwide. Colistin and tigecycline are considered as the last resort drugs against carbapenem-resistant bacteria (Cunha et al., 2017; Zhou Y. et al., 2019). Either the global distribution of colistin-resistant *E. coli* or the rapid spread of the carbapenem-resistant *Enterobacteriaceae* have created enormous challenges for public health security. It is a more and more headache to solve the infection caused by MDR pathogens in human clinical treatment and animal husbandry (Gao et al., 2016; Potter et al., 2016; Rehman et al., 2020; Zhang et al., 2021b, 2021c). As a result, tigecycline has been recognized as the important antibiotic of last resort for the clinical treatment of certain bacterial infections. Through this article, we found that the *tet*(X) is prevalent on six continents around the world, with China having the highest prevalence, and most of *tet*(X4)-carrying plasmids can spread tigecycline resistance among different bacteria by means of horizontal transfer.

The mechanisms that cause antibiotic resistance to tigecycline are mainly overexpression of active efflux pump and ribosomal protection mechanisms. However, more and more *tet*(X4) has been detected in plasmids, and many different types of *tet*(X4)-carrying plasmids have strong ability of horizontal transfer, which means plasmids mediated transmission of tigecycline resistance genes may gradually increase, risking to public health (Pereira et al., 2021). The widespread use of antimicrobial drugs in domestic animals is an important reason for the rapid increase of AMR. The researchers reported the AMR monitoring results of *E. coli* in China’s pig farms from 2018 to 2019, showing that multidrug resistance was detected in 91% of isolates (1871 in total), and resistance to last resort drugs including tigecycline, colistin and carbapenem was found (Peng et al., 2022). Recent studies have also found the antibiotic resistance of livestock has increased from 1970 to 2019, indicating that if the use of antibiotics is not restricted, it may not be able to effectively protect the livestock. By testing the sensitivity of several recent strains of *E. coli* to various antibiotics, researchers found their resistance was far higher than that of the strains in the 1970s. In addition, the researchers also pointed out although the specific antibiotics used to treat bacterial infections may be different, the types are often the same, so the rapid rise in drug resistance will eventually affect human beings (Yang et al., 2022). Surprisingly, the potential spread of virus-mediated ARGs is likely to exacerbate AMR, including tetracycline resistance and harm to public health (Calero-Cáceres et al., 2019; Debroas and Siguret, 2019; Shi et al., 2022), which needs our wider attention. Moreover, viruses might be linked to *Enterobacteriaceae* or *Vibrionaceae* and were considered as gene shuttles in ARGs transfer, like plasmids. This indicates that viruses and bacteria may have a synergistic effect on the transmission of ARGs. Therefore，we should look at AMR from a holistic perspective that includes humans, animals as well as the environment, and develop a plan for rational use of antibiotics to reduce the long-term and single use of tigecycline in the clinical environment, avoiding reduced clinical efficacy and increased mortality (Yahav et al., 2011). Controlling the “spillover effect” of ARGs is also important from “One Health” concept (Collignon, 2015; Tyrrell et al., 2019; Olesen et al., 2020; Aslam et al., 2021). In-depth studies of tigecycline resistance or transmission mechanisms, and continuous monitoring of *tet*(X) prevalence are urgent needed to determine the precise transmission route of ARB and ARGs, so as to provide reference for designing more effective public health intervention strategies. However, due to the limitation of the length of the article, we did not summarize the current methods and strategies of various countries or regions to limit the transmission of *tet*(X4)-positive strains, and what beneficial substances (like probiotics, prebiotics and antimicrobial peptide) can replace use of specific antibiotics in the post-antibiotic era to avoid the spread of tigecycline resistance.

## Author contributions

SZ and JW wrote this manuscript. JW, YuW, and SZ contributed to the design of this manuscript. MW, XO, QY, YiW, RJ, ML, DZ, SC, and QG provided ideas for the conception of this manuscript. BT, DS, XZ, SM, and JH helped to create figures and tables. SZ and AC modified this manuscript, and acquired funding. All authors contributed to the article and approved the submitted version.

## Funding

This work was supported by the NSFC (31902267), the earmarked fund for China Agriculture Research System (CARS-42-17), the funds of Key Laboratory of Livestock and Poultry Provenance Disease Research in Mianyang, Sichuan Veterinary Medicine and Drug Innovation Group of China Agricultural Research System (SCCXTD-2022-18), and the Applied Basic Research Programs of Science and Technology Department of Sichuan Province (2019YJ0410).

## Conflict of interest

The authors declare that the research was conducted in the absence of any commercial or financial relationships that could be construed as a potential conflict of interest.

## Publisher’s note

All claims expressed in this article are solely those of the authors and do not necessarily represent those of their affiliated organizations, or those of the publisher, the editors and the reviewers. Any product that may be evaluated in this article, or claim that may be made by its manufacturer, is not guaranteed or endorsed by the publisher.

## Footnotes

^1^ http://faculty.washington.edu/marilynr/
